# Drug-Induced Tubulointerstitial Nephritis: Insights From the World Health Organization Safety Database

**DOI:** 10.1016/j.ekir.2022.04.090

**Published:** 2022-05-05

**Authors:** Alexandre O. Gérard, Diane Merino, Audrey Laurain, Marion Cremoni, Marine Andreani, Fanny Rocher, Alexandre Destere, Vincent L.M. Esnault, Antoine Sicard, Milou-Daniel Drici

**Affiliations:** 1 Department of Nephrology-Dialysis-Transplantation, University Hospital Centre of Nice, Nice, France; 2 Department of Pharmacology and Pharmacovigilance Center of Nice, University Hospital Centre of Nice, Nice, France; 3 Laboratory of Molecular Physio Medicine (LP2M), UMR 7370, Centre National de le Recherche Scientifique, University Côte d’Azur, Nice, France; 4 Department of Immunology, University Hospital Centre of Nice, Nice, France; 5 Clinical Research Unit of University Côte d’Azur (UR2CA), University Côte d’Azur, Nice, France

**Keywords:** allergy, database, drugs, nephrotoxicity, pharmacovigilance, tubulointerstitial nephritis

## Introduction

Tubulointerstitial nephritis (TIN) is characterized by renal failure associated with an array of histologic lesions relating to an infiltration of inflammatory cells in the kidney interstitium with edema, often accompanied by tubulitis.^1^ The histologic pattern can also evolve into fibrosis, especially if TIN is left undiagnosed. TIN can arise from infections or autoimmune diseases (lupus, Sjögren, sarcoidosis, IgG4-related disease, etc.), but the most common cause remains an immune-mediated reaction to drugs, accounting for more than two-thirds of cases.^2^ Drug-induced TIN accounts for 3% to 15% of renal biopsy samples.^3^^,^^4^ This number increases up to 27% when only patients with unexplained acute renal failure are considered.^5^

The presumptive diagnosis is often challenging, given the heterogeneous clinical picture. TIN is typically characterized by a (sub)acute rise in creatininemia, which is seldom specific.^6^ Besides, there is often no abnormality in the urinalysis. Therefore, anamnesis is pivotal to the early diagnosis. Etiologic investigations should rely on a thorough knowledge of the most suspect drugs, to promptly discontinue the possible culprit. Furthermore, early treatment with steroids may speed renal recovery.^5^

Yet, quantitative and qualitative data about the association of TIN with various drugs are scarce. Therefore, we aimed to rank the drugs having the strongest association with TIN, based on the international pharmacovigilance database and a disproportionality analysis.

## Results

### Characteristics of the Reports

Between November 14, 1967, and August 31, 2021, a total of 15,696 cases of TIN (Preferred Term from the Medical Dictionary for Regulatory Activities, MedDRA) were reported in VigiBase (Supplementary Material for Methods).S23-S29 The most represented age group was the 45- to 64-year-old group with 4075 reports (35.3%), and the median age was 60 years (interquartile range: 43–71). Men were involved in 6881 reports (53.1%). The United States of America contributed 7959 reports (50.7%). Notifiers were health care professionals in 9667 reports (61.6%). Detailed characteristics are provided in Table 1.

**Table 1:** Characteristics of the reports of patients with tubulointerstitial nephritis

Characteristics	Number of reports (%)
Age	
≤23 mo	52 (0.5)
2–11 yr	235 (2.0)
12–17 yr	554 (4.8)
18–44 yr	2143 (18.6)
45–64 yr	4075 (35.3)
65–74 yr	2459 (21.3)
≥75 yr	2030 (17.8)
Country	
United States	7959 (50.7)
Japan	1251 (8.0)
United Kingdom	990 (6.3)
France	972 (6.2)
Australia	705 (4.5)
Spain	520 (3.3)
Germany	484 (3.1)
Canada	437 (2.8)
Sweden	257 (1.6)
Switzerland	231 (1.5)
Reporter qualification	
Physician	6062 (38.6)
Pharmacist	1487 (9.5)
Other health professional	2118 (13.5)
Lawyer	1175 (7.5)
Consumer	1551 (9.9)
Unknown	3671 (23.4)

### Characteristics of the TIN

Among 15,696 reports, 12,481 (79.5%) were deemed serious, including 977 fatal cases (6.2%). The median time to onset was 20 (interquartile range: 5–103) days. The MedDRA terms most frequently co-reported were acute kidney injury, in 6350 reports (40.5%), and chronic kidney disease in 4292 cases (27.3%). Pyrexia (512 reports, 3.3%), nausea (391 reports, 2.5%), rash (375 reports, 2.4%), and eosinophilia (320 reports, 2.0%) were the most frequently coreported extrarenal terms. Among 8221 cases with an available outcome, TIN completely recovered in 3019 reports (36.7%), did not recover in 2676 reports (32.6%), and was recovering or recovered with sequelae (without returning to baseline kidney function) in 2526 reports (30.7%).

### Active Ingredients Ranked by Absolute Number of Reports

Most of the reported drugs belong to the “alimentary tract and metabolism” and the “antiinfectives for systemic use” categories (Anatomical Therapeutic Chemical classification system), with 8204 (52.3%) and 4525 (28.8%) notifications, respectively. Specifically, proton pump inhibitors (PPIs) were the most frequently reported drugs, with 5769 reports (36.8%). Omeprazole was the most reported (4328, 27.6%), followed by lansoprazole and esomeprazole. Aside from PPIs, the most reported active ingredient was ciprofloxacin with 529 reports (3.4%), followed by ibuprofen (497, 3.2%), vancomycin (430, 2.7%), diclofenac (299, 1.9%), and sulfamethoxazole/trimethoprim (292, 1.9%). As a whole, β-lactams accounted for 1461 reports.

### Disproportionality Analysis

In the disproportionality analysis, 223 active ingredients were characterized by a positive IC025. PPI (as a whole: reporting odds ratio [ROR] 57.3; 95% CI 55.5–59.2) reached the 9 strongest ROR, including dexlansoprazole (ROR 209.9; 95% CI 200.0–220.3), lansoprazole (ROR 134.1; 95% CI 129.1–139.3), and rabeprazole (ROR 127.3; 95% CI 119.6–135.4), *inter alia*. Penicillin antibiotics, such as nafcillin (ROR 63.2; 95% CI 51.0–78.3) and flucloxacillin (ROR 31.4; 95% CI 27.3–36.0), were also characterized by strong ROR. As a whole, β-lactam was characterized by a ROR of 4.8 (95% CI 4.6–5.1). Other drugs significantly associated with TIN included sodium phosphate (ROR 55.6; 95% CI 45.3–68.4) and mesalazine (ROR 35.0; 95% CI 31.6–38.8). The ranking of active ingredients with IC025 > 0 and ≥30 reports is provided in Table 2.

**Table 2:** Active ingredients disproportionately reported (IC025 >0) with tubulointerstitial nephritis, ranked by number of reports (only the 15 most reported drugs are displayed, see Supplementary Table S1 for a more comprehensive listing)

Active ingredient	Number of reports (%)	ROR (95% CI)
Omeprazole	4328 (27.6)	84.5 (81.6–87.6)
Lansoprazole	3571 (22.8)	134.1 (129.1–139.3)
Esomeprazole	3532 (22.5)	79.6 (76.7–82.7)
Pantoprazole	3185 (20.3)	100 (96.2–104.1)
Dexlansoprazole	2067 (13.2)	209.9 (200–220.3)
Rabeprazole	1142 (7.3)	127.3 (119.6–135.4)
Ciprofloxacin	529 (3.4)	7.9 (7.3–8.7)
Ibuprofen	497 (3.2)	5.3 (4.9–5.8)
Vancomycin	430 (2.7)	10.8 (9.8–11.9)
Piperacillin/tazobactam	399 (2.5)	11.8 (10.7–13.1)
Mesalazine	387 (2.5)	35 (31.6–38.8)
Esomeprazole/naproxen	309 (2)	64.4 (57.5–72.2)
Diclofenac	299 (1.9)	3.5 (3.2–4)
Nivolumab	293 (1.9)	9.7 (8.6–10.9)
Sulfamethoxazole/trimethoprim	292 (1.9)	4.2 (3.7–4.7)

ROR, reporting odds ratio.

## Discussion

Although virtually any drug can be associated with a risk of TIN, this analysis of the World Health Organization safety database highlights the most frequently involved. These drugs should alert the clinician facing suspicion of TIN or acute renal failure of undetermined cause. PPIs, antibiotics, and nonsteroidal anti-inflammatory drugs are the most reported drugs for TIN. This ranking is confirmed by the disproportionality analysis, which is a more reliable indicator of the comparative risk for a drug to be associated with TIN.

Previous studiesS1–S3 suggested that TIN was more frequent in the elderly. Indeed, their reduced renal function makes them prone to the accumulation of drugs and metabolites. They are also exposed to a wider range of drugs. In VigiBase, half of the reports concerned patients > 60 years of age. Half of the reported TINs occurred during the first 3 weeks of treatment, whereas 1 quarter occurred after >3 months. Thus, the time to onset may be slightly longer than previously described.S3^,^S4 In our retrospective study, approximately one-third of patients recovered completely, which is in the lower range of what has been previously reported (between one- and two-thirds of complete recoveries).^6^^,^S5^,^S6 Besides, >1 quarter of the patients ended up with chronic kidney disease.

As expected,S6^,^S7 PPIs account for more than one-third of TIN reports in VigiBase. The magnitude of their reporting is likely subsequent to their wide use, exposing a large share of the population to their adverse effects. Their strong association with TIN stands out even in the disproportionality analysis, which includes the total number of adverse drug reactions notified with a given drug.

In line with previous studies,S2^,^S8 antibiotics are also frequently reported, especially fluoroquinolones (e.g., ciprofloxacin) and β-lactams. Some are widely used, such as amoxicillin, mechanically increasing the absolute number of reports. Conversely, barely used β-lactams, such as nafcillin, are associated with a strong disproportionality signal. Regarding vancomycin, there is a signal regarding its association with immune-allergic TIN, beyond the well-known cases of acute tubular necrosis, possibly associated with tubular casts.S9–S12

The association of nonsteroidal anti-inflammatory drugs (e.g., ibuprofen) with TIN was foreseeable.^6^^,^S6^,^S13–S15 Their ROR may be underestimated, because they are involved in many other renal and extrarenal adverse drug reactions. As for immune checkpoint inhibitors (e.g., nivolumab), their association with TIN is confirmed, even though the exact underlying mechanism is still debated.S16–S20

Sodium phosphate can elicit acute or chronic phosphate nephropathy,S21 which pathophysiology is totally different from common drug-induced TIN (of immunoallergic mechanism).

To our knowledge, this study about TIN is the first to rely on a comprehensive analysis of >15,000 reports from the World Health Organization safety database, with drugs used in a real-life setting. Though, pharmacovigilance studies are hindered by under-reporting.S22 Conversely, we cannot exclude that some diagnoses of TIN might have been wrongly attributed to the notified drug. A confirmation of the diagnosis by renal biopsy may have lacked in some cases, as these data are not provided in VigiBase. In fact, incomplete data are inherent to pharmacovigilance studies and hinder thorough qualitative assessments of the reports. For instance, the rate of recovery may have been slightly higher if patients classified as recovering had been followed longer. The MedDRA terminology, used in VigiBase, prevented us from fully discriminating between acute and chronic forms of TIN. Besides, clinicians are prone to ascribe a TIN diagnosis to a drug already known to cause TIN (reporting bias). Thus, to partly mitigate this potential confirmation bias, the ranking according to the absolute number of reports was underpinned by a disproportionality analysis. The large sample size may partly compensate the qualitative heterogeneity of data. Overall, those pharmacovigilance approaches can only suggest and compare safety signals and do not allow to conclude on a causal association between an effect and a given drug.

In this comprehensive analysis of the World Health Organization safety database, a ranking of drugs associated with TIN has been established. The strongest associations with TIN involve PPIs, antibiotics (β-lactams, fluoroquinolones, glycopeptides), and nonsteroidal anti-inflammatory drugs. Early identification of a potential causative drug is pivotal to increase chances of recovery. Hence, pharmacovigilance data can foster the efficient management of (sub)acute renal failure of undetermined origin.

## Disclosure

All the authors declared no competing interests.

## Supplementary Material

**Supplementary Methods.**

**Supplementary References.**

**Table S1.** Active ingredients disproportionately reported (IC025 > 0) with tubulointerstitial nephritis, ranked by number of reports (only active ingredients involved in ≥30 reports are displayed).
