# Supplementary material for: Drug-Induced Tubulointerstitial Nephritis: Insights From the World Health Organization Safety Database
Source: Kidney Int Rep. 2022 May 5;7(7):1699–702. doi: 10.1016/j.ekir.2022.04.090 (PMC9263231; doi:10.1016/j.ekir.2022.04.090)
Supplement: Supplementary File (PDF) [file mmc1.pdf]

SUPPLEMENTARY MATERIAL

METHODS

Data source

The Uppsala Monitoring Centre (UMC) is mandated by the World Health Organization (WHO) to monitor drug safety since 1978. UMC collates data from each national pharmacovigilance network of 172 member countries, so that VigiBase® includes all data issued from these networks. Spontaneous and voluntary notifications from healthcare professionals, non-healthcare professionals as well as pharmaceutical companies are collected into VigiBase®, respecting the anonymity of both patients and notifiers. Collected data include sociodemographic characteristics (age, sex, notifier's country) and details about active ingredients (suspect, interacting or concomitant active ingredients, start and end of treatment, indication, dosage regimen) and the reported effect (type of ADR, co-reported terms, date of occurrence, seriousness).

Query

VigiBase® was queried for all reports of TIN registered between November 14, 1967 (first reports in VigiBase®) and August 31, 2021. TIN was defined by the "tubulointerstitial nephritis" Preferred Term (PT) from the Medical Dictionary for Regulatory Activities (MedDRA, version 24.0). MedDRA is a clinically validated international medical terminology dictionary used by regulatory authorities. The PT represents a distinct descriptor for a single medical concept.[S23] Characteristics of reports were described in terms of medians (with

interquartile range) for quantitative variables, and in terms of effectives and proportions for qualitative ones.

Disproportionality analysis

Thereupon, potential drug safety signals of interest were investigated using two methods of disproportionality analysis, namely the Reporting Odd Ratio (ROR) and the Information Component (IC), for the combination of each active ingredient with TIN. This method has also been used and described elsewhere.[S24-S27]

ROR approximates the odds ratio of case-control studies and is specific to case-non-case studies. It helps in estimating the extent to which a given ADR is associated with a specific active ingredient. The ROR is the odds of exposure to each active ingredient among TIN reports divided by the odds of exposure to all the other drugs recorded in the database among all others ADRs reports. A ROR of 1 translates as no signal: the ADR is similarly reported with the drug of interest and the comparator. Conversely, a ROR > 1 suggests an ADR more frequently reported with the drug of interest. The higher the ROR, the more statistically relevant is the pharmacovigilance signal. The ROR was expressed as a point estimate with a 95% Confidence Interval (CI), using Woolf's method.

IC is derived from a Bayesian confidence propagation neural network[S28] and reflects the strength of the association of an active ingredient with an ADR.[S29] IC lowers the risk of false-positive signals, especially when a given ADR has a very low expected frequency in the database (artificially increasing the ROR). IC025 is the lower end of the 95% CI of the IC. A positive IC025 is the traditional threshold used in statistical signal detection at UMC.

Therefore, IC025 was used to identify meaningful associations. Then, active ingredients characterized with a significant association with TIN were ranked according to their absolute

number of TIN reports. ROR was used to further assess the strength of each association. The formulas of ROR and IC are described below.

	TIN	Other ADRs
Selected active ingredient	A	B
All other active ingredients	C	D

ROR = (A/C)/(B/D)

IC = log2 [(A + 0.5)/($N_{expected}$ + 0.5)] where $N_{expected}$ = [(A + B) × (A + C)]/(A + B + C + D)

SUPPLEMENTARY REFERENCES

S1. Goicoechea M, Rivera F, López-Gómez JM, Spanish Registry of Glomerulonephritis. Increased prevalence of acute tubulointerstitial nephritis. *Nephrol Dial Transplant Off Publ Eur Dial Transpl Assoc - Eur Ren Assoc*. 2013;28(1):112-115. doi:10.1093/ndt/gfs143

S2. Oyama S, Hosohata K, Inada A, et al. Drug-induced tubulointerstitial nephritis in a retrospective study using spontaneous reporting system database. *Ther Clin Risk Manag*. 2018;14:1599-1604. doi:10.2147/TCRM.S168696

S3. Davison AM, Jones CH. Acute interstitial nephritis in the elderly: a report from the UK MRC Glomerulonephritis Register and a review of the literature. *Nephrol Dial Transplant Off Publ Eur Dial Transpl Assoc - Eur Ren Assoc*. 1998;13 Suppl 7:12-16. doi:10.1093/ndt/13.suppl_7.12

S4. Caillard S, Moulin B. Néphropathie interstitielle immuno-allergiqueDrug-induced acute interstitial nephritis. *Réanimation*. 2003;12(4):306-312. doi:10.1016/S1624-0693(03)00060-4

S5. González E, Gutiérrez E, Galeano C, et al. Early steroid treatment improves the recovery of renal function in patients with drug-induced acute interstitial nephritis. *Kidney Int*. 2008;73(8):940-946. doi:10.1038/sj.ki.5002776

S6. Muriithi AK, Leung N, Valeri AM, et al. Biopsy-proven acute interstitial nephritis, 1993-2011: a case series. *Am J Kidney Dis Off J Natl Kidney Found*. 2014;64(4):558-566. doi:10.1053/j.ajkd.2014.04.027

S7. Al-Aly Z, Maddukuri G, Xie Y. Proton Pump Inhibitors and the Kidney: Implications of Current Evidence for Clinical Practice and When and How to Deprescribe. *Am J Kidney Dis*. 2020;75(4):497-507. doi:10.1053/j.ajkd.2019.07.012

S8. Muriithi AK, Leung N, Valeri AM, et al. Clinical characteristics, causes and outcomes of acute interstitial nephritis in the elderly. *Kidney Int*. 2015;87(2):458-464. doi:10.1038/ki.2014.294

S9. Codding CE, Ramseyer L, Allon M, Pitha J, Rodriguez M. Tubulointerstitial Nephritis Due to Vancomycin. *Am J Kidney Dis*. 1989;14(6):512-515. doi:10.1016/S0272-6386(89)80152-0

S10. Hsu SI. Biopsy-proved acute tubulointerstitial nephritis and toxic epidermal necrolysis associated with vancomycin. *Pharmacotherapy*. 2001;21(10):1233-1239. doi:10.1592/phco.21.15.1233.33901

S11. Bergman MM, Glew RH, Ebert TH. Acute interstitial nephritis associated with vancomycin therapy. *Arch Intern Med*. 1988;148(10):2139-2140.

S12. Tantranont N, Luque Y, Hsiao M, et al. Vancomycin-Associated Tubular Casts and Vancomycin Nephrotoxicity. *Kidney Int Rep*. 2021;6(7):1912-1922. doi:10.1016/j.ekir.2021.04.035

S13. Henao J, Hisamuddin I, Nzerue CM, Vasandani G, Hewan-Lowe K. Celecoxib-induced acute interstitial nephritis. *Am J Kidney Dis*. 2002;39(6):1313-1317. doi:10.1053/ajkd.2002.33412

S14. Praga M, González E. Acute interstitial nephritis. *Kidney Int*. 2010;77(11):956-961. doi:10.1038/ki.2010.89

S15. Bennett WM, Henrich WL, Stoff JS. The renal effects of nonsteroidal anti-inflammatory drugs: Summary and recommendations. *Am J Kidney Dis*. 1996;28(1):S56-S62. doi:10.1016/S0272-6386(96)90570-3

S16. Cassol C, Satoskar A, Lozanski G, et al. Anti–PD-1 Immunotherapy May Induce Interstitial Nephritis With Increased Tubular Epithelial Expression of PD-L1. *Kidney Int Rep*. 2019;4(8):1152-1160. doi:10.1016/j.ekir.2019.06.001

S17. Shingarev R, Glezerman IG. Kidney Complications of Immune Checkpoint Inhibitors: A Review. *Am J Kidney Dis*. 2019;74(4):529-537. doi:10.1053/j.ajkd.2019.03.433

S18. Gérard AO, Andreani M, Fresse A, et al. Immune checkpoint inhibitors-induced nephropathy: a French national survey. *Cancer Immunol Immunother CII*. Published online June 21, 2021. doi:10.1007/s00262-021-02983-8

S19. Tawhari I, Fenton SE, Sosman JA, Sustento-Reodica N, Kanwar YS, Aggarwal V. Hyperacute Onset of Immune Checkpoint Inhibitor–Related Acute Interstitial Nephritis. *Kidney Int Rep*. 2020;5(11):2084-2088. doi:10.1016/j.ekir.2020.08.002

S20. Jhaveri KD, Wanchoo R, Sakhiya V, Ross DW, Fishbane S. Adverse Renal Effects of Novel Molecular Oncologic Targeted Therapies: A Narrative Review. *Kidney Int Rep*. 2017;2(1):108-123. doi:10.1016/j.ekir.2016.09.055

S21. Markowitz GS, Perazella MA. Acute phosphate nephropathy. *Kidney Int*. 2009;76(10):1027-1034. doi:10.1038/ki.2009.308

S22. Hazell L, Shakir SAW. Under-reporting of adverse drug reactions : a systematic review. *Drug Saf*. 2006;29(5):385-396. doi:10.2165/00002018-200629050-00003

S23. MedDRA Hierarchy | MedDRA. Accessed September 3, 2021. https://www.meddra.org/how-to-use/basics/hierarchy

S24. Salem JE, Manouchehri A, Moey M, et al. Cardiovascular toxicities associated with immune checkpoint inhibitors: an observational, retrospective, pharmacovigilance study. *Lancet Oncol*. 2018;19(12):1579-1589. doi:10.1016/S1470-2045(18)30608-9

S25. Mahé J, de Campaigno EP, Chené AL, Montastruc JL, Despas F, Jolliet P. Pleural adverse drugs reactions and protein kinase inhibitors: Identification of suspicious targets by

disproportionality analysis from VigiBase. *Br J Clin Pharmacol*. 2018;84(10):2373-2383. doi:10.1111/bcp.13693

S26. Bai X, Lin X, Zheng K, et al. Mapping endocrine toxicity spectrum of immune checkpoint inhibitors: a disproportionality analysis using the WHO adverse drug reaction database, VigiBase. *Endocrine*. 2020;69(3):670-681. doi:10.1007/s12020-020-02355-9

S27. Hennessy S. Disproportionality analyses of spontaneous reports. *Pharmacoepidemiol Drug Saf*. 2004;13(8):503-504. doi:10.1002/pds.995

S28. Bate A, Lindquist M, Edwards IR, et al. A Bayesian neural network method for adverse drug reaction signal generation. *Eur J Clin Pharmacol*. 1998;54(4):315-321. doi:10.1007/s002280050466

S29. UMC | Analytics in VigiLyze. Accessed September 3, 2021. https://www.who-umc.org/vigibase/vigilyze/analytics-in-vigilyze/

SUPPLEMENTARY TABLE

Active ingredients disproportionately reported (IC025>0) with tubulointerstitial nephritis, ranked by number of reports (only active ingredients involved in ≥30 reports are displayed)

CI: Confidence Interval; ROR: Reporting Odds Ratio

Active ingredient	Number of reports (%)	ROR (95% CI)
**Omeprazole**	4,328 (27.6)	84.5 (81.6-87.6)
**Lansoprazole**	3,571 (22.8)	134.1 (129.1-139.3)
**Esomeprazole**	3,532 (22.5)	79.6 (76.7-82.7)
**Pantoprazole**	3,185 (20.3)	100 (96.2-104.1)
**Dexlansoprazole**	2,067 (13.2)	209.9 (200-220.3)
**Rabeprazole**	1,142 (7.3)	127.3 (119.6-135.4)
**Ciprofloxacin**	529 (3.4)	7.9 (7.3-8.7)
**Ibuprofen**	497 (3.2)	5.3 (4.9-5.8)
**Vancomycin**	430 (2.7)	10.8 (9.8-11.9)
**Piperacillin/Tazobactam**	399 (2.5)	11.8 (10.7-13.1)
**Mesalazine**	387 (2.5)	35 (31.6-38.8)
**Esomeprazole/Naproxen**	309 (2)	64.4 (57.5-72.2)
**Diclofenac**	299 (1.9)	3.5 (3.2-4)
**Nivolumab**	293 (1.9)	9.7 (8.6-10.9)
**Sulfamethoxazole/Trimethoprim**	292 (1.9)	4.2 (3.7-4.7)
**Levofloxacin**	242 (1.5)	2.6 (2.2-2.9)
**Amoxicillin**	229 (1.5)	3 (2.6-3.4)
**Tacrolimus**	227 (1.4)	5.6 (4.9-6.4)
**Amoxicillin/Clavulanic acid**	221 (1.4)	2.6 (2.3-2.9)
**Pembrolizumab**	215 (1.4)	12 (10.4-13.7)
**Paracetamol**	210 (1.3)	2.1 (1.9-2.5)
**Flucloxacillin**	206 (1.3)	31.4 (27.3-36)
**Allopurinol**	206 (1.3)	9.4 (8.2-10.8)
**Mycophenolic acid**	193 (1.2)	5.7 (5-6.6)
**Rifampicin**	184 (1.2)	5.8 (5-6.7)
**Loxoprofen**	172 (1.1)	15.8 (13.6-18.4)

**Furosemide**	160 (1)	4.2 (3.6-4.9)
**Ceftriaxone**	158 (1)	1.7 (1.4-2)
**Naproxen**	143 (0.9)	2.2 (1.8-2.6)
**Clozapine**	138 (0.9)	1.4 (1.2-1.7)
**Omeprazole/Sodium bicarbonate**	131 (0.8)	114.4 (95.8-136.6)
**Fluindione**	129 (0.8)	9.8 (8.2-11.7)
**Prednisone**	125 (0.8)	2.7 (2.3-3.2)
**Cefepime**	122 (0.8)	16.3 (13.6-19.5)
**Tenofovir**	122 (0.8)	6 (5-7.1)
**Gentamicin**	118 (0.8)	9.4 (7.8-11.3)
**Clarithromycin**	111 (0.7)	3.8 (3.1-4.5)
**Ipilimumab**	105 (0.7)	7.8 (6.4-9.5)
**Lithium**	101 (0.6)	6 (4.9-7.3)
**Ciclosporin**	100 (0.6)	2.9 (2.4-3.6)
**Azithromycin**	99 (0.6)	2.4 (1.9-2.9)
**Sodium phosphate**	94 (0.6)	55.6 (45.3-68.4)
**Levetiracetam**	91 (0.6)	3.4 (2.8-4.2)
**Cloxacillin**	89 (0.6)	7.4 (6-9.1)
**Celecoxib**	89 (0.6)	1.9 (1.5-2.3)
**Metformin**	88 (0.6)	1.6 (1.3-2)
**Nafcillin**	87 (0.6)	63.2 (51-78.3)
**Zoledronic acid**	85 (0.5)	2.6 (2.1-3.3)
**Emtricitabine/Tenofovir**	84 (0.5)	4 (3.2-4.9)
**Carbamazepine**	81 (0.5)	2 (1.6-2.5)
**Clindamycin**	79 (0.5)	1.9 (1.5-2.4)
**Amlodipine**	79 (0.5)	1.4 (1.1-1.7)
**Prednisolone**	76 (0.5)	2 (1.6-2.5)
**Meropenem**	69 (0.4)	5.4 (4.2-6.8)
**Valproic acid**	69 (0.4)	1.4 (1.1-1.8)
**Mefenamic acid**	66 (0.4)	6 (4.7-7.7)
**Rofecoxib**	66 (0.4)	1.8 (1.4-2.2)
**Aciclovir**	64 (0.4)	4.4 (3.4-5.6)
**Hydrochlorothiazide**	64 (0.4)	3.9 (3-5)

**Cefalexin**	63 (0.4)	4.2 (3.3-5.3)
**Moxifloxacin**	63 (0.4)	2.4 (1.8-3)
**Methylprednisolone**	63 (0.4)	2.2 (1.8-2.9)
**Rosuvastatin**	62 (0.4)	1.7 (1.3-2.2)
**Lisinopril**	60 (0.4)	1.7 (1.3-2.2)
**Dicloxacillin**	58 (0.4)	8.4 (6.5-10.9)
**Enalapril**	58 (0.4)	1.8 (1.4-2.3)
**Ketoprofen**	57 (0.4)	3 (2.3-3.9)
**Cefazolin**	57 (0.4)	2.2 (1.7-2.8)
**Basiliximab**	54 (0.3)	27.2 (20.8-35.6)
**Ampicillin**	54 (0.3)	2.5 (1.9-3.3)
**Daptomycin**	52 (0.3)	11.8 (9-15.5)
**Metamizole**	52 (0.3)	3.3 (2.5-4.4)
**Lamotrigine**	52 (0.3)	1.4 (1.1-1.8)
**Doxycycline**	51 (0.3)	2.4 (1.8-3.1)
**Azathioprine**	50 (0.3)	2.8 (2.1-3.7)
**Ritonavir**	47 (0.3)	4.6 (3.5-6.2)
**Famotidine**	46 (0.3)	4.5 (3.4-6)
**Indometacin**	46 (0.3)	3.4 (2.6-4.6)
**Cimetidine**	46 (0.3)	3 (2.2-4)
**Cefuroxime**	46 (0.3)	1.4 (1.1-1.9)
**Antithymocyte immunoglobulin**	45 (0.3)	7.5 (5.6-10)
**Benzylpenicillin**	44 (0.3)	1.5 (1.1-2)
**Minocycline**	41 (0.3)	4.9 (3.6-6.6)
**Sulfasalazine**	41 (0.3)	2.5 (1.8-3.4)
**Ifosfamide**	40 (0.3)	4.2 (3-5.7)
**Ketorolac**	40 (0.3)	2.3 (1.7-3.1)
**Atazanavir**	39 (0.2)	7.4 (5.4-10.2)
**Tobramycin**	39 (0.2)	4.9 (3.6-6.7)
**Pemetrexed**	39 (0.2)	2.9 (2.1-4)
**Lamivudine**	38 (0.2)	2.7 (2-3.7)
**Amiodarone**	38 (0.2)	1.4 (1-2)
**Indinavir**	37 (0.2)	8.3 (6-11.5)

**Rebamipide**	37 (0.2)	6 (4.3-8.2)
**Valaciclovir**	37 (0.2)	4.6 (3.3-6.3)
**Aristolochia fontanesii**	36 (0.2)	299.9 (210.6-427.1)
**Tosufloxacin**	36 (0.2)	95.5 (68.3-133.6)
**Meloxicam**	36 (0.2)	3.1 (2.3-4.4)
**Sitagliptin**	35 (0.2)	2 (1.4-2.8)
**Ceftazidime**	35 (0.2)	1.9 (1.4-2.6)
**Leflunomide**	34 (0.2)	2 (1.4-2.8)
**Nitrofurantoin**	33 (0.2)	2.3 (1.7-3.3)
**Ramipril**	32 (0.2)	1.6 (1.1-2.3)
**Piperacillin**	31 (0.2)	8.7 (6.1-12.4)
**Febuxostat**	30 (0.2)	8.1 (5.6-11.5)
**Losartan**	30 (0.2)	1.9 (1.3-2.7)
**Ethambutol**	30 (0.2)	1.5 (1.1-2.2)
